# Metabolic Phenotyping Predicts Gemcitabine and Cisplatin Chemosensitivity in Patients With Cholangiocarcinoma

**DOI:** 10.3389/fpubh.2022.766023

**Published:** 2022-02-10

**Authors:** Manida Suksawat, Jutarop Phetcharaburanin, Poramate Klanrit, Nisana Namwat, Narong Khuntikeo, Attapon Titapun, Apiwat Jarearnrat, Vanlakhone Vilayhong, Prakasit Sa-ngiamwibool, Anchalee Techasen, Arporn Wangwiwatsin, Panupong Mahalapbutr, Jia V. Li, Watcharin Loilome

**Affiliations:** ^1^Department of Biochemistry, Faculty of Medicine, Khon Kaen University, Khon Kaen, Thailand; ^2^Cholangiocarcinoma Research Institute, Khon Kaen University, Khon Kaen, Thailand; ^3^Khon Kaen University International Phenome Laboratory, Northeastern Science Park, Khon Kaen University, Khon Kaen, Thailand; ^4^Cholangiocarcinoma Screening and Care Program (CASCAP), Khon Kaen University, Khon Kaen, Thailand; ^5^Department of Surgery, Faculty of Medicine, Khon Kaen University, Khon Kaen, Thailand; ^6^Department of Pathology, Faculty of Medicine, Khon Kaen University, Khon Kaen, Thailand; ^7^Faculty of Associated Medical Sciences, Khon Kaen University, Khon Kaen, Thailand; ^8^Department of Metabolism, Digestion and Reproduction, Faculty of Medicine, Imperial College London, South Kensington Campus, London, United Kingdom

**Keywords:** cholangiocarcinoma (CCA), gemcitabine, cisplatin, nuclear magnetic resonance (NMR) spectroscopy, predictive biomarker

## Abstract

Gemcitabine and cisplatin serve as appropriate treatments for patients with cholangiocarcinoma (CCA). Our previous study using histoculture drug response assay (HDRA), demonstrated individual response patterns to gemcitabine and cisplatin. The current study aimed to identify predictive biomarkers for gemcitabine and cisplatin sensitivity in tissues and sera from patients with CCA using metabolomics. Metabolic signatures of patients with CCA were correlated with their HDRA response patterns. The tissue metabolic signatures of patients with CCA revealed the inversion of the TCA cycle that is evident with increased levels of citrate and amino acid backbones as TCA cycle intermediates, and glucose which corresponds to cancer stem cell (CSC) properties. The protein expression levels of CSC markers were examined on tissues and showed the significantly inverse association with the responses of patients to cisplatin. Moreover, the elevation of ethanol level was observed in gemcitabine- and cisplatin-sensitive group. In serum, a lower level of glucose but a higher level of methylguanidine was observed in the gemcitabine-responders as non-invasive predictive biomarker for gemcitabine sensitivity. Collectively, our findings indicate that these metabolites may serve as the predictive biomarkers in clinical practice which not only predict the chemotherapy response in patients with CCA but also minimize the adverse effect from chemotherapy.

## Introduction

The worldwide highest incidence of cholangiocarcinoma (CCA), the bile duct cancer which originates from bile duct epithelial cells, is found in Thailand, especially the northeastern region, where it is recognized as one of the major public health problems (1). The development of CCA is associated with the liver fluke, *Opisthorchis viverrini* (Ov), infection, which is defined as the major risk factor of CCA development (2). Moreover, the majority of CCA cases are diagnosed at an advanced or metastasis stage due to the clinical silence in the early stages of the disease (3). Therefore, curative surgery and adjuvant chemotherapy are required to improve the survival of patients with CCA (4, 5). According to the advanced biliary cancer (ABC) guidelines, gemcitabine and a combination of gemcitabine and cisplatin serve as the appropriate treatment for patients with CCA (6). The response pattern of patients to chemotherapy is the one of key factors in chemotherapy success (7). However, our previous study demonstrated that patients with CCA have their own individual response pattern to gemcitabine and cisplatin based on the histoculture drug response assay (HDRA) method, and the majority of patients with CCA seems to be non-responsive to both gemcitabine and cisplatin (8). Therefore, biomarkers for predicting the gemcitabine and cisplatin chemosensitivity are urgently required.

The development of biomedical research field has moved from the study of the genome, transcriptome, and proteome to the metabolome (9). The study of metabolome or metabolomics measures a set of low-molecular-weight metabolites, which provide a snapshot of the final products of cellular processes, at a time point under a specific environmental stimulus (10–12). To date, especially in the field of cancer research, metabolomics is also applied to the discovery of potential biomarkers, for example, for early detection of ovarian (13) and colon cancer (14). In addition, colorectal cancer metabolomics offered an analytical platform to predict the toxicity of capecitabine (15). In chemosensitivity, it can be used to predict the neoadjuvant chemotherapy response in breast cancer (16) as well as the platinum-based combination chemotherapy response in lung cancer (17). The metabolic signatures provide information on the pathological condition, the molecular mechanisms, and predictive/diagnostic biomarkers, which can be of considerable clinical benefit.

In the current study, a ^1^H nuclear magnetic resonance (NMR) spectroscopy-based metabolomics was used to identify predictive biomarkers for gemcitabine and cisplatin sensitivity in both tumor tissues and serum samples of patients with CCA.

## Patients And Methods

### Patients and Sample Collection

The serum samples, prior to surgery, and surgically resected CCA tumor tissues were obtained from patients who were diagnosed with CCA and had undergone surgery at Srinagarind Hospital, Khon Kaen University, Thailand. The written inform consent was obtained from all patients. The protocol for specimen collection was approved by the Ethics Committee for Human Research, Khon Kaen University (HE571283, HE591330, HE601149, and HE611576) and all the studies were performed in accordance with relevant guidelines and regulations. The tissue samples from patients with CCA were divided into three parts, which were described in our previous study (8). The first part was submitted into HDRA and for the second part, the tissues were fixed with 10% formalin and embedded in paraffin for immunohistochemical staining. For the last part, the tissues were stored at −80°C for NMR-based metabolomics experiment. The serum samples of patients with CCA were stored at −80° C until the NMR-based metabolomics experiment started. The clinicopathological data of each patient were provided by the Cholangiocarcinoma Research Institute (CARI), Khon Kaen University and the Cholangiocarcinoma Screening and Care Program (CASCAP), Khon Kaen University. Each patient was defined into responder and non-responder subgroups according to their response pattern to treatments from the HDRA method.

### Sample Preparation

For metabolomics analysis, tumor tissues were weighed to approximately 100 mg for each case and washed with 1x phosphate-buffered saline (PBS) pH 7.4 prior to the extraction with 400 μl methanol and chloroform (v:v, 1:1) mixture. After centrifugation at 10,000 g for 15 min, the mixture was separated into a polar phase and a lipophilic phase. The solvents were removed using a speed vacuum concentrator (Labconco, Kansas City, MO, USA). The tissue extracts were resuspended in high-performance liquid chromatography (HPLC)-grade water (100 mg of tissue: 600 μl of HPLC water) and vortexed until the extracts completely dissolved. Then, the samples were centrifuged at 18,000 g for 10 min at 4°C. A total of 540 μl of supernatant was transferred to new tube and mixed with 60 μl of the buffer containing 100% D_2_O, 1.5 M KH_2_PO_4_, 2 mM NaN_3_, and 1% of 3-trimethysilypropionic acid (TSP) (Cambridge Isotype Laboratories, Tewksbury, MA, USA). TSP peak was used to calibrate the spectra with a chemical shift reference (δ^1^H = 0 ppm). Then, the sample was briefly spun down and a total of 580 μl of supernatant was transferred to a 5 mm NMR tube. Serum samples were defrosted at room temperature and spun at 18,000 g, at 4°C for 10 min. Then, a total of 300 μl of supernatant from each sample was transferred to an Eppendorf tube and mixed with 300 μl of plasma buffer containing 0.075 M Na_2_HPO_4_, 2 mM NaN_3_, and 0.08% TSP solution (pH 7.4). The mixture was briefly spun down and a total of 580 μl of supernatant was transferred to a 5 mm NMR tube. The entire serum preparation process was performed on ice and was randomized.

### ^1^H Nuclear Magnetic Spectra Processing and Metabolite Identification

The ^1^H NMR spectra were acquired using a 600 MHz NMR spectrometer (Bruker, Germany) at 300 K for tissue extracts and 310 K for serum samples using a published protocol (18). A standard 1-dimension pulse sequence (cycle decay-90°-t_1_-90°-t_m_-90°-acquisition) and 64 scans were used. The NMR spectra were phased, calibrated, and baseline corrected automatically in Topspin software (version 2.1). The water peak (4.5–5.0 ppm) and TSP (−1 to 0.551 ppm) region were removed. Then, the metabolic data of tissues were processed through the alignment (19) and normalization (20). Whereas, the serum's data were processed with alignment. Principal component analysis (PCA) and orthogonal partial-least square discriminant analysis (O-PLS-DA) were performed using SIMCA-P+ version 15 (Umetrics Inc., Sweden). The significance of model was calculated by permutation in MATLAB (R2015a). The data were mean-centered and Pareto scaled. The metabolite identification was confirmed using statistical total correlation spectroscopy (STOCSY) in MATLAB against the database which include in-house database, ChenomxNMR Suite analysis, and Human Metabolome Database (HMBD). The list of metabolites that found in tumor tissues and serum are presented in Supplementary Tables S1, S2, respectively. Additionally, the crucial metabolites discriminating the responder from the non-responder group were quantified based on the area under the peak.

### Antibodies

Antibodies to EpCAM (1:100 dilution), CD133 (1:100 dilution), CD44V6 (1: 50 dilution), and ALDH1A1 (1:50 dilution) were purchased from Abcam (Abcam, Cambridge, United Kingdom). Antibody to CD44v8-10 (1:50 dilution) was purchased from Cosmo Bio (Cosmo Bio, Tokyo, Japan).

### Immunohistochemistry

For the immunohistochemistry (IHC) staining, we followed the previous standard protocol for immunoperoxidase staining (21). The sections of human CCA tissues were deparaffinized and rehydrated through xylene and an increasing series of ethanol solutions. Then, antigen retrieval was performed in sodium citrate plus Tween 20 buffer for 10 min using a microwave. Next, endogenous peroxidase activity was blocked with 0.3% hydrogen peroxide in PBS for 30 min. Furthermore, 10% skim milk in PBS was used to block the non-specific binding for 1 h. The tissue sections were then incubated with the primary antibody against the designated target proteins overnight at 4°C. Then, the tissue sections were washed in 0.1% (v/v) Tween-20 in PBS for 5 min (three times) followed by PBS for 5 min (once) and incubated with peroxidase-conjugated Envision secondary antibody (DAKO, Denmark, K4003) for 1 h at room temperature. The slides were then washed with 0.1% (v/v) Tween-20 in PBS for 5 min (three times) followed by PBS for 5 min (once). Further, a 3,3'-diaminobenzidine (DAB) solution was used to develop color in the sections for 5–10 min. The sections were then counterstained with Mayer's hematoxylin. Finally, the sections were dehydrated in a series of ethanol solutions of increasing concentration followed by xylene and then mounted. The results of immunohistochemical staining of human CCA tissues were quantified based on the scoring system which depended on the intensity and frequency of staining in tumor area. The intensity of staining was defined into three groups: 1+, weak staining; 2+, moderate staining; and 3+, strong staining. The frequency of staining was defined in four groups: 0% = negative; 1+, 1–25%; 2+, 26–50%; and 3+, >50%. The multiplying of intensity and frequency is staining score in each case, and the median score for all cases was calculated. The results were classified into two groups: in the low expression group, the score was less than the median and in the high expression group, the score was equal to or greater than the median.

### Statistical Analysis

Statistical analysis was performed using SPSS software version 17 (IBM Corporation, NY, USA). The difference among the response and non-response pattern to chemotherapy was analyzed using *t*-tests. The data was illustrated as a graph of mean ± SD, using Graph Pad prism 5 (GraphPad Software, Inc., CA, USA). Moreover, the associations between the expression of proteins in human CCA tissue and the *in vitro* method were determined by Fisher's exact test. All the analyses were two-tailed and a *p* < 0.05 was considered statistically significant.

## Results

### Patient Characteristics

The characteristics of from patients with CCA whom the metabolic signatures of the tumor tissues were studied are summarized in Table 1. The characteristics of patients with CCA whom the serum sample analyzed using the NMR spectroscopy are shown in Table 2. The majority of patients with CCA were men with a median age of 63 years old. Intrahepatic with non-papillary histology and margin free status were present in the majority of cases. At the time of diagnosis, most of patients with CCA were at the stages III and IV.

**Table 1:** The characteristics of all patients with cholangiocarcinoma (CCA) whose tumor tissues were studied by metabolomics profiling.

**Variables**	**Number (n)**
**Sex (*****n*** **=** **36)**	
Male	23
Female	13
**Age (year)**	
Less than 61	15
61 or greater	21
**Tumor site**	
Intrahepatic	20
Extrahepatic	16
**Histology**	
Papillary	9
Non-papillary	27
**Marginal status**	
Free margin	17
Not free margin	11
Not applicable	8
**Primary tumor (T)**	
Is, I, II	11
III, IV	19
Not applicable	6
**Reginal lymph node (N) metastasis**	
No	14
Yes	8
Not applicable	14
**Distance metastasis**	
No	14
Yes	6
Not applicable	16
**TNM stage**	
I, II	9
III, IV	22
Stage unknown	5

**Table 2:** The characteristics of all patients with CCA whose serum was studied by metabolomics profiling.

**Variables**	**Number (n)**
**Sex**	
Male	21
Female	13
**Age (year)**	
Less than 61	14
61 or greater	20
**Tumor site**	
Intrahepatic	19
Extrahepatic	15
**Histology**	
Papillary	8
Non-papillary	26
**Marginal status**	
Free margin	16
Not free margin	11
Not applicable	7
**Primary tumor (T)**	
Is, I, II	10
III, IV	19
Not applicable	5
**Reginal lymph node (N) metastasis**	
No	13
Yes	8
Not applicable	13
**Distance metastasis (M)**	
No	13
Yes	5
Not applicable	16
**TNM stage**	
I, II	8
III, IV	22
Stage unknown	4

### Pattern Recognition of ^1^H NMR Metabolic Profiles and Chemotherapy Response Patterns in Tissues of Patients With CCA

**Figure 1:**
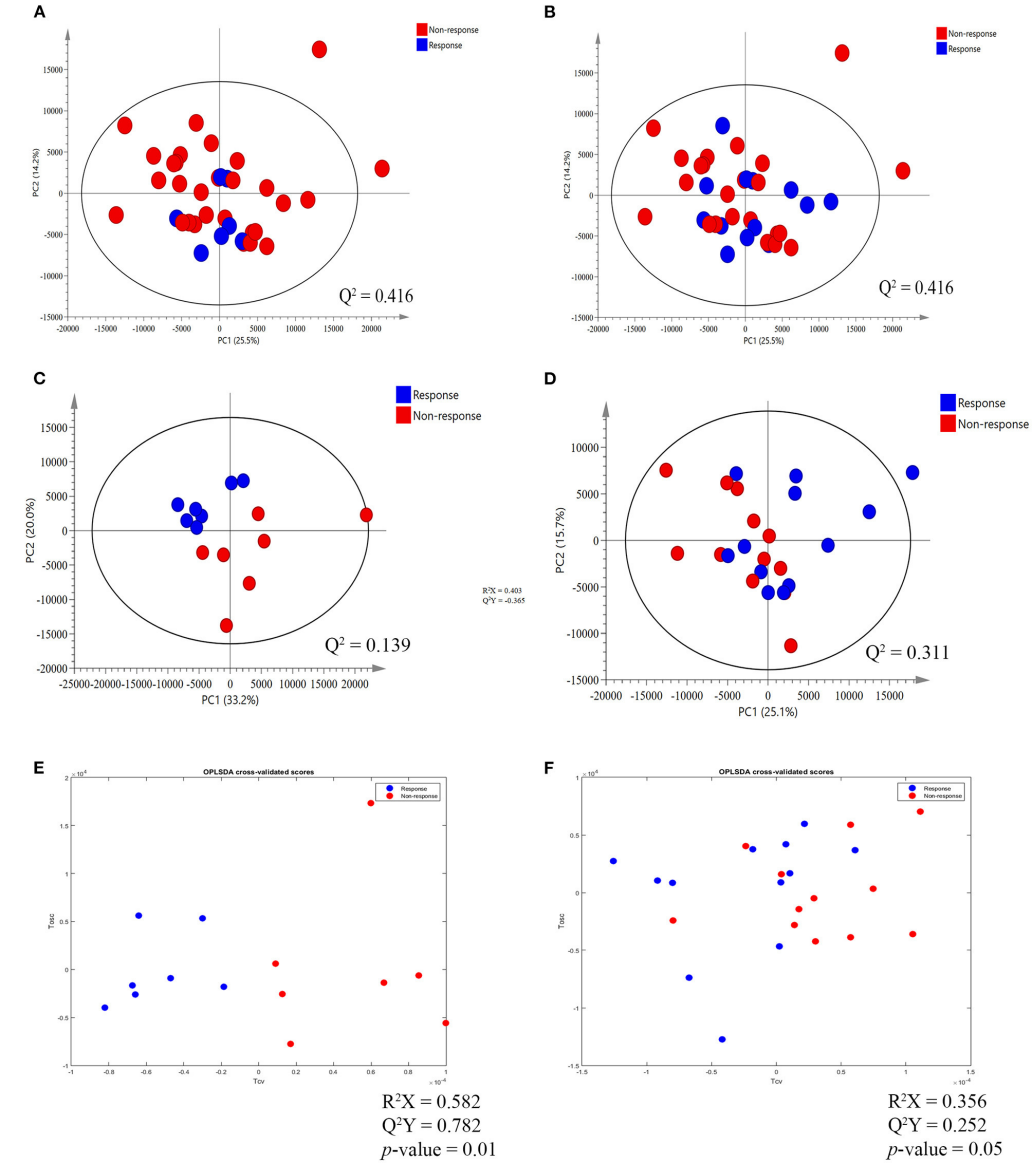
Principal component analysis (PCA) score plot and orthogonal partial-least square discriminant analysis (O-PLS-DA) cross validated score plot between response and non-response to gemcitabine and cisplatin in tumor tissues of patients with cholangiocarcinoma (CCA). **(A,B)** The PCA score plot between response and non-response group to gemcitabine and cisplatin, respectively, before match the meta-data of patients with CCA. **(C,D)** The PCA score plot, after match the meta-data of patients with CCA, between response and non-response group to gemcitabine and cisplatin, respectively. **(E,F)** The O-PLS-DA cross validated score plot between the response and non-response group to gemcitabine and cisplatin, respectively. The data were Pareto scaled.

**Figure 2:**
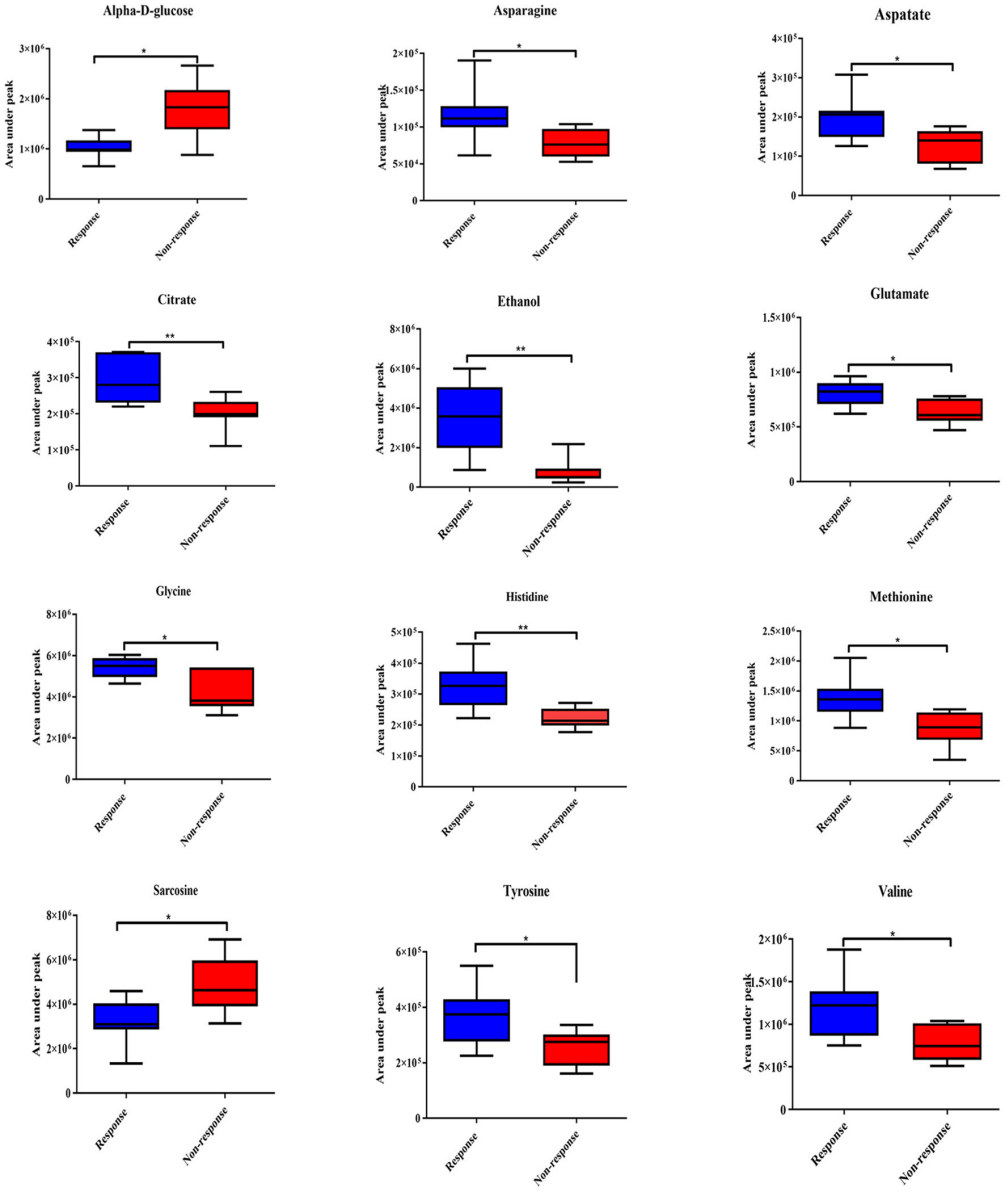
Significantly changed metabolites in gemcitabine response patterns from tumor tissues of patients with CCA. The blue color shows response group and red color shows non-response group. * and ** indicate statistically significant p-value <0.05 and *p*-value <0.01, respectively.

**Figure 3:**
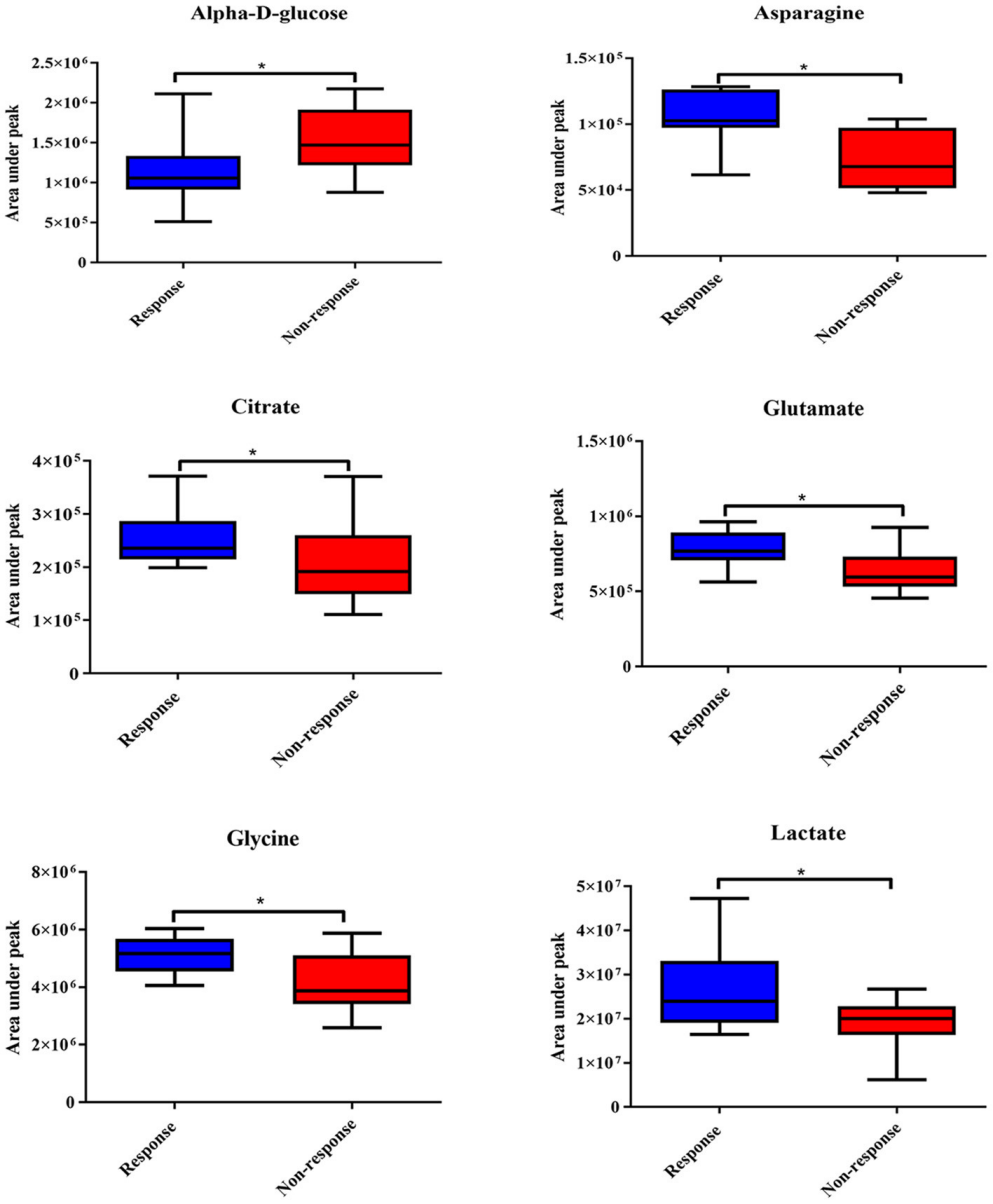
Significantly changed metabolites in cisplatin response patterns from tumor tissues of patients with CCA. The blue color shows response group and red color shows non-response group. * indicates statistically significant.

The ^1^H NMR metabolic signatures from the tumor tissues of patients with CCA are represented in Supplementary Figure S1. To detect outliers and visualize the clustering of the metabolic profiles of patients between response and non-response patterns, unsupervised PCA was conducted. PCA score plots (Figures 1A,B) were color coded based on the responses to either gemcitabine or cisplatin, and there was no clear clustering observed in the model with the entire sample set. As human cohort studies often contain confounding factors (22), we then sub-sampled employing sex- and age-matched patients with CCA from the responder and non-responder groups and carried out the PCA analysis (Figures 1C,D for gemcitabine and cisplatin, respectively). The results clearly revealed that responder and non-responder groups of patients with CCA can be distinguished by the first two principal components for gemcitabine, whereas no clear clustering was observed in cisplatin. Supervised O-PLS-DA models were calculated to discriminate metabolic profiles of responders and non-responders (Figures 1E,F for gemcitabine and cisplatin, respectively). The significantly higher levels of asparagine, aspartate, citrate, glycine, histidine, ethanol, methionine, and valine, and the lower levels of alpha-D-glucose in the tumor tissues were observed in the gemcitabine drug-responders in contrast to the non-responders (Figure 2). With regard to the responses to cisplatin, the levels of asparagine, citrate, glutamate, glycine, and lactate significantly decreased in the non-responders compared with the responders (Figure 3). Interestingly, the level of alpha-D-glucose greatly increased in non-responders to cisplatin. Higher levels of asparagine, citrate, and glycine and lower levels of alpha-D-glucose were observed in responders to both gemcitabine and cisplatin compared with the non-responders. Higher levels of ethanol were also observed in responders to cisplatin, but did not reach the statistical significance.

### Expression Level of CSC Markers in Tumor Tissues of Patients With CCA

**Figure 4:**
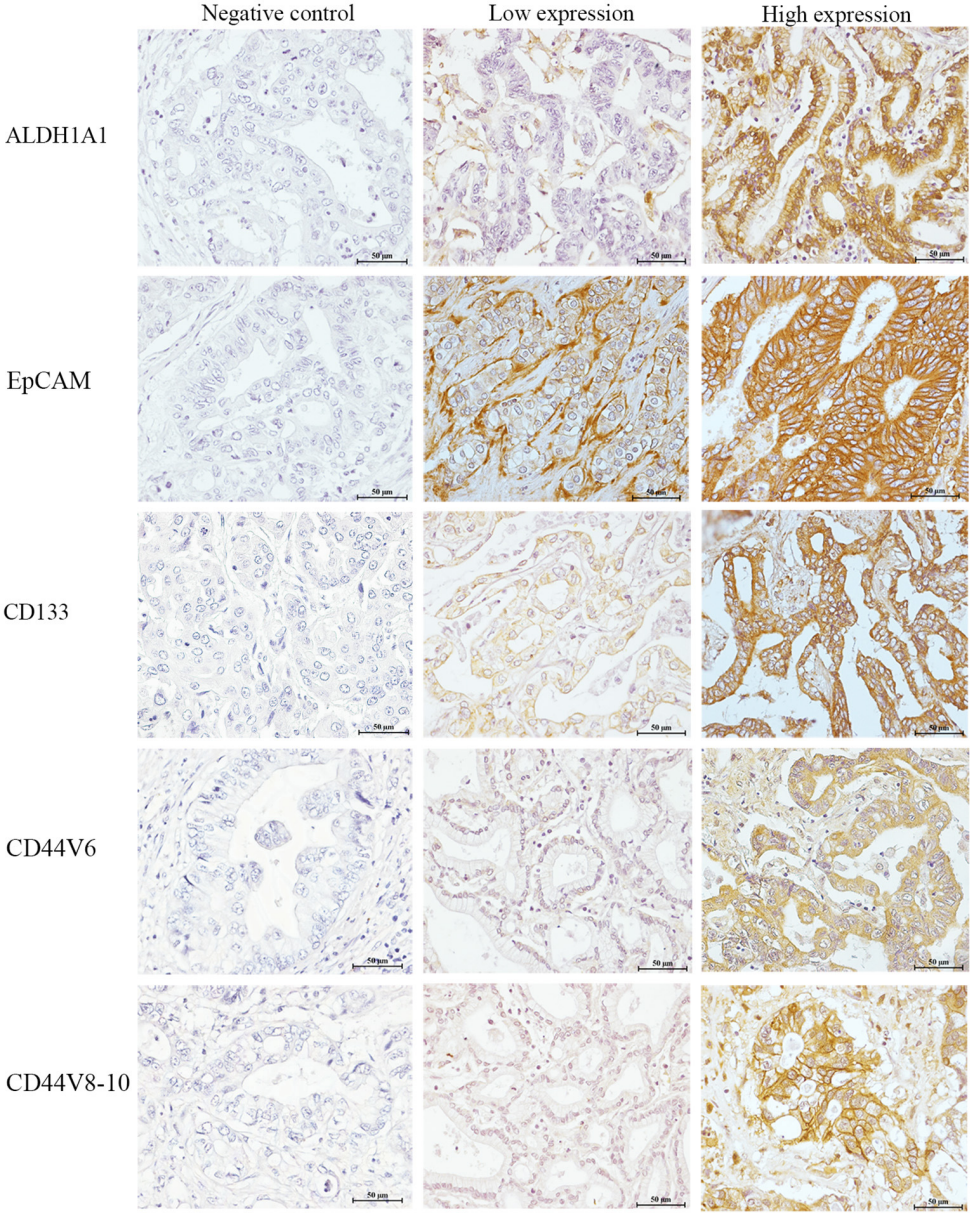
The expression of cancer stem cell (CSC) markers in human CCA tissues. The left panel represents the negative control. The middle panel represents low expression of each CSC marker. The right panel represents high expression of each CSC marker. Bar 50 μm (insert).

Based on our metabolomics study, there was an elevation of the alpha-D-glucose level but a substantial decrease in the level of metabolites involved in tricarboxylic acid (TCA) cycle of gemcitabine and cisplatin non-responders. This is consistent with the signature of CSCs (23). We, therefore, further hypothesized that the tumor tissues of patients with CCA who showed no response to both gemcitabine and cisplatin may contain a higher population of CSCs than those who responded to both chemotherapeutic drugs. The expression levels of CSC markers, such as ALDH1A1, EpCAM, CD133, CD44V6, and CD44V8-10, were determined in the tumor tissues through IHC staining (Figure 4).

The correlation of the expression level of CSC markers and response patterns to gemcitabine and cisplatin was analyzed. The results showed that the high expressions of ALDH1A1, EpCAM, and CD44V6 were significantly associated with the non-response pattern to cisplatin (*p* = 0.041, *p* = 0.017, and *p* = 0.014, respectively). Additionally, high expressions of CD133 and CD44V8-10 tended to be associated with the non-response pattern to cisplatin, while the high expressions of ALDH1A1, EpCAM, CD133, CD44V6, and CD44V8-10 showed a trend to be positively correlated with the non-response pattern to gemcitabine significance which was not reached (Table 3).

**Table 3:** Correlation of the expression levels of cancer stem cell (CSC) markers with the gemcitabine and cisplatin response pattern from histoculture drug response assay (HDRA) results.

**Expression levels**		**Gemcitabine response patterns**			**Cisplatin response patterns**		
		**Response (*n* = 7)**	**Non-response (*n* = 29)**	***p*-value**	**Response (*n* = 12)**	**Non-response (*n* = 24)**	***p*-value**
ALDH1A1	High	1	16	0.092	3	14	0.041^*^
	Low	6	13		10	9	
EpCAM	High	2	19	0.103	6	17	0.017^*^
	Low	5	10		9	4	
CD133	High	4	17	1.000	9	14	0.736
	Low	3	12		6	7	
CD44V6	High	2	17	0.219	3	16	0.014^*^
	Low	5	12		10	7	
CD44V8-10	High	3	18	0.418	8	15	0.310
	Low	4	11		7	6	

* *p < 0.005, statistically significant*.

### Pattern Recognition of ^1^H Serum NMR Metabolic Profiles in Response to Chemotherapies

**Figure 5:**
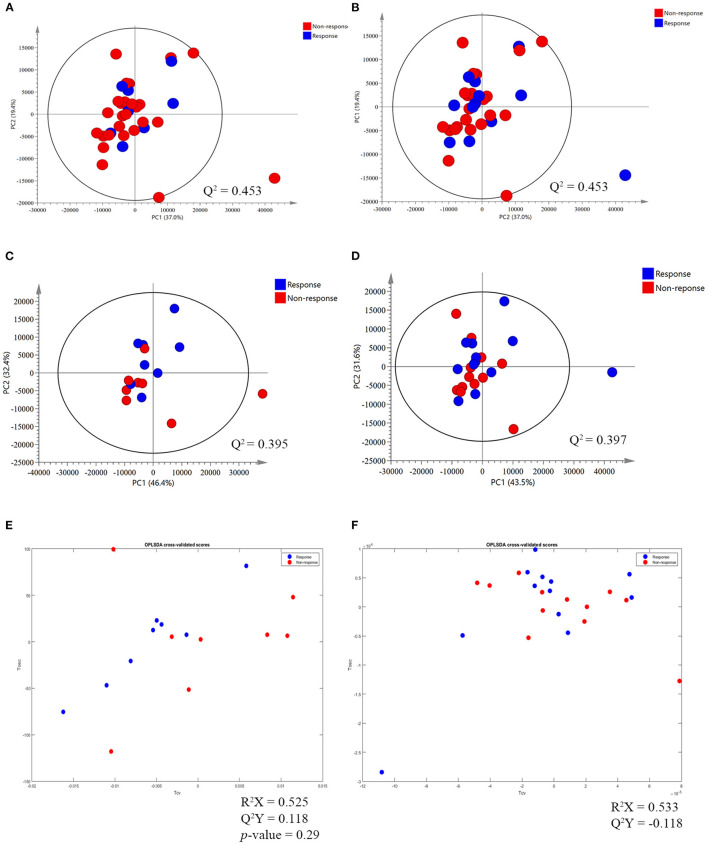
The PCA and O-PLS-DA cross validated score plot between response and non-response patterns of gemcitabine and cisplatin in serum sample of patients with CCA. **(A,B)** The PCA score plot between the response and non-response group to gemcitabine and cisplatin, respectively, before matching the meta-data of patients with CCA. **(C,D)** The PCA score plot between the response and non-response group to gemcitabine and cisplatin, respectively, after matching the meta-data. **(E,F)** O-PLS-DA cross validated score plot between the response and non-response group to gemcitabine and cisplatin, respectively. The data were Pareto scaled.

**Figure 6:**
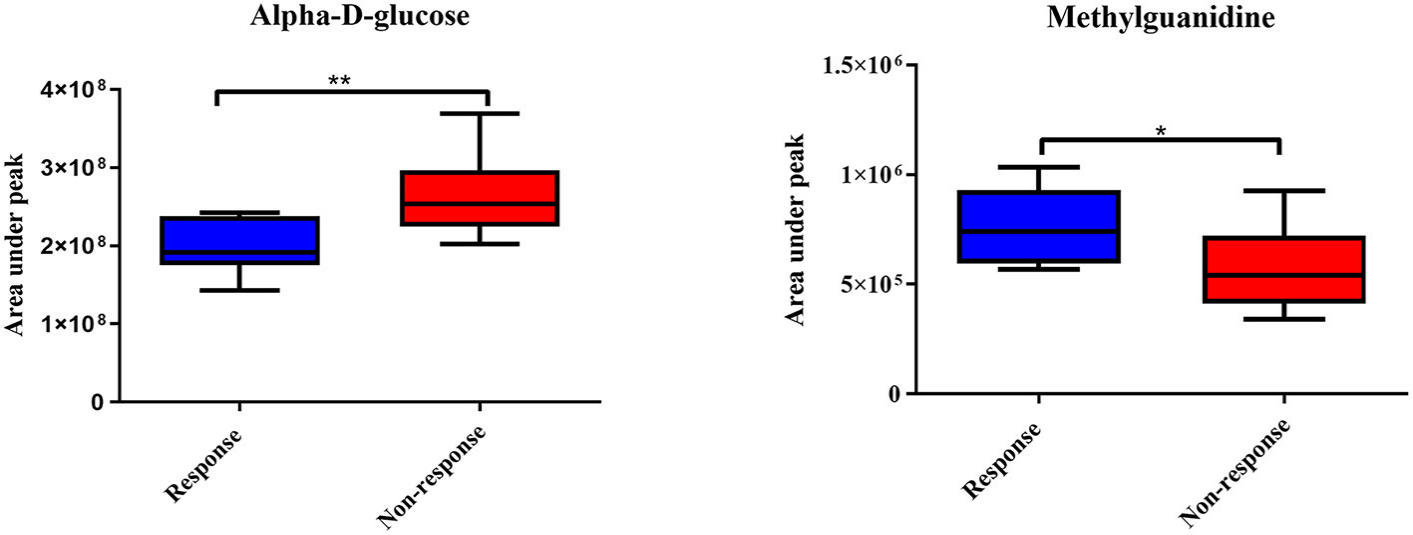
Significantly changed metabolites in gemcitabine response patterns from serum samples of patients with CCA. The blue color shows the response group and red color shows the non-response group. * and ** indicate statistically significant *p*-value < 0.05 and *p*-value < 0.01, respectively.

Apart from the tumor tissues of patients with CCA, we aimed to identify the predictive biomarkers of gemcitabine and cisplatin sensitivity in serum samples of patients with CCA. The representative ^1^H NMR spectra of serum samples are shown in Supplementary Figure S3. No clear patterns were observed between responders and non-responders in PCA scores plots, both before and after sex- and age-matched (Figures 5A–D), and no significant O-PLS-DA cross validated models were derived from the two groups either (Figures 5E,F). The response patterns of gemcitabine showed a significant increase in the level of methylguanidine in patients who responded to gemcitabine. Interestingly, the level of alpha-D-glucose increased in the group of patients who showed no response to gemcitabine, in good agreement with the result from tissue samples (Figure 6).

## Discussion

Here, we first identify the predictive biomarkers for gemcitabine and cisplatin chemosensitivity at the metabolite level in the tissues and serum of patients with CCA using an NMR-based metabolic profiling. The patients were divided into responder and non-responder subgroups according to their HDRA response patterns to gemcitabine and cisplatin. The correlation of individual metabolic signatures and their response patterns to chemotherapies was analyzed. In metabolome data analysis, we sub-sampled employing sex- and age-matched from responder and non-responder groups to avoid the influence of sex-age dependency which was defined as confounding factor and deeply affect both qualitative and quantitative analysis of metabolites. From the previous study on adult human plasma metabolome that showed age-associated changes of the relative concentration of more than 100 metabolites. In addition, effect of sex-associated as well as the influence of sex on age-associated changes in metabolites was previously reported (24). Moreover, the influence of sex in urinary children metabolome was reported of which the changes of metabolites in sex difference groups were still present even with the age stratification (25). After sex- and age-matched, patients who had no response to both gemcitabine and cisplatin demonstrated similar fingerprints. The significantly higher level of glucose and lower levels of amino acids, which serve as carbon sources of TCA cycle intermediates (26), found in the non-response group for both chemotherapies, suggesting that gemcitabine and cisplatin non-responded may possess the low activity in the TCA cycle. Interestingly, the increased glucose level in non-responders was reported in lung cancer (17). The upregulation of glucose level was found in the serum of patients with lung cancer who were insensitive to platinum-based combination chemotherapy. Additionally, the high level of glucose in CCA was associated with CCA progression as shown in the previous *in-vitro* study (27). The evidence of sarcosine in the chemotherapeutic response has not been widely studied. However, in prostate cancer, sarcosine is defined as an oncometabolite involved in cancer development and progression (28, 29). In addition, the response to cisplatin was lactate dependent. Lactate is a major energy source in tumor growth, resulting in the increased cancer cell proliferation (30). Thus, it serves as a target in cisplatin activity. Therefore, the high level of lactate is associated with a response pattern to cisplatin. Moreover, we demonstrated that tumor tissues of patients with CCA contain endogenous ethanol which may play a crucial role in chemosensitivity. The endogenous ethanol was believed to originate from the microbial fermentation in the gastro-intestinal tract (31–33). Correspondingly, nowadays, the accumulation of data suggested that not only the gastro-intestinal bacteria but also the intratumoral bacteria can show the ability to modulate chemotherapeutic drug gemcitabine and cisplatin response (34–37). Therefore, the endogenous ethanol found in tumor tissues of patients with CCA may originate from intratumoral bacteria and the insolvent of the intratumoral bacteria in chemotherapeutic drug response in CCA may shed new light and need to be further investigated.

From the metabolic phenotypes of non-responders to either chemotherapy exhibiting the lowered TCA cycle activity with high level of glucose were consistent with the signature of CSCs which was reported in several previous studies (23, 38–41). In brain tumor, the CSCs reveal their signature through the low activity of oxidative phosphorylation (41). In term of glucose, it can induce the expression of specific gene in CSC associated with glucose metabolism, such as *c-Myc, PDK-1* which results in the increased CSC population (39). Moreover, the increase of glucose uptake was found in CSC compared with non-CSC (38, 40). We further hypothesized that the patients who are non-responders to gemcitabine and cisplatin may contain, in their tumor tissues, a greater population of CSCs than those who respond to gemcitabine and cisplatin. The expression levels of CSC markers, such as ALDH1A1, EpCAM, CD133, CD44V6, and CD44V8-10, were confirmed in the tumor tissues of patients with CCA using IHC staining. This phenomenon was correlated with the HDRA-based response patterns of patients with CCA. The high expressions of ALDH1A1, EpCAM, and CD44V6 were significantly associated with the non-response pattern of cisplatin, whereas high expressions of CD133 and CD44V8-10 were likely correlated with non-response to cisplatin. Apart from this, the high expressions of all CSC markers tended to be associated with non-response patterns to gemcitabine but did not reach significance, which may due to the low number of patients who responded to gemcitabine. These results correspond to the previous study from our group, which found the overexpression of CSC markers in CCA tissues. Moreover, the overexpression of the CSC markers can serve as an indicator for CCA recurrence (42).

Apart from the tumor tissues, we examined the serum of patients with CCA for predictive biomarkers of gemcitabine and cisplatin responses. In the gemcitabine responder group, alpha-D-glucose was increased in patients who did not response to gemcitabine. In addition, a significant decrease in methylguanidine was found in this group. Methylguanidine is the metabolic production of creatinine as well as some amino acids (43, 44). In pancreatic cancer, methylguanidine serves as a predictive biomarker for early detection (45). Unfortunately, the direct role of methylguanidine in chemotherapy response has yet to be reported. The study of chronic renal failure, however, shows that methylguanidine can induce apoptosis of renal proximal tubular cells through the inhibition of antioxidant effect (46). Likewise, in our study, methylguanidine may potentially promote gemcitabine sensitivity by inhibiting the antioxidants. In the cisplatin responders, the levels of alpha-D-glucose and methylguanidine were found to follow the similar trend; however, the supervised model of cisplatin response was not significant.

**Figure 7:**
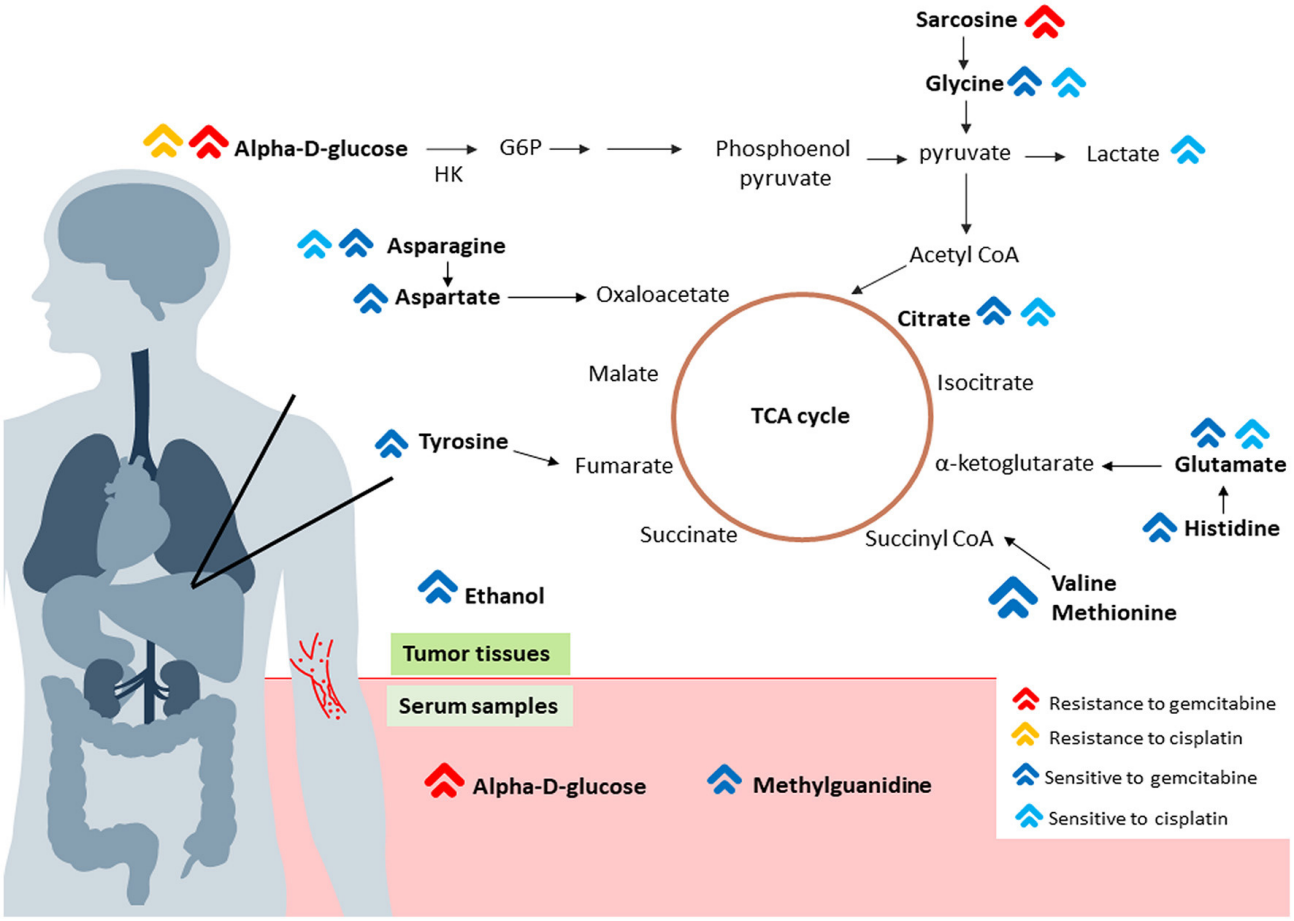
The possible schematic diagram of the metabolic pathways in both tumor tissues and serum samples of patients with CCA. The red arrows represent metabolite high in a group of patients who are resistant or non-responsive to gemcitabine. The yellow arrow represents metabolite high in a group of patients who are non-responsive to cisplatin. On the other hand, the dark blue arrows represent metabolite high in a group of patients who are responsive to gemcitabine. The blue arrows represent metabolite high in a group of patients who responded to cisplatin. The schematic indicated that, tumor tissues, the low activity of TCA cycles with high glucose in a group of patients who are non-responsive to both gemcitabine and cisplatin consistent with CSC signature. Moreover, high glucose level found in serum sample of patients who are non-responsive to gemcitabine and high level of methylguanidine was found in group of patients who are responsive to gemcitabine.

In the light of this study, individual response patterns of patients with CCA to gemcitabine and cisplatin are dependent on the population of CSCs in their tumor tissues which is represented by the inversion of TCA cycle and glucose level. The remarkable increase in the citrate level together with amino acids backbones to produce intermediates for TCA cycle were found in gemcitabine and cisplatin-sensitive group compared with the insensitive counterpart while the low level of glucose was accompanied. Apart from that, the other intermediates of TCA cycle, such as fumarate, malate, and succinate seem to increase in gemcitabine and cisplatin-sensitive group despite the invalid statistical models which may arise from the inter-variation among patients with CCA and small sample size. Moreover, ethanol influences the response pattern of patients with CCA to gemcitabine and cisplatin. Therefore, the level of alpha-D-glucose, TCA cycle metabolites along with ethanol may serve as predictive biomarkers for gemcitabine and cisplatin chemosensitivity for patients with CCA. Moreover, methylguanidine may serve as the serum predictive biomarker for gemcitabine sensitivity. Altogether, the possible mechanistic biochemical pathways in both tumor tissue and serum samples of patients with CCA using NMR-based metabolomics study are illustrated in Figure 7. The Figure 7 was generated using Microsoft PowerPoint version 2019 (https://www.microsoft.com/th-th/microsoft-365/get-started-with-office-2019).

Our study may lead to clinical advantages not only for the choice of chemotherapy but also for the maximized effect of chemotherapy in individual patients with CCA. Nevertheless, the association of these predictive biomarkers with the clinical chemotherapeutic response needs to be further validated. Especially, the biomarker at the metabolite level need to go through five phases of validation (47). The translation of biomarker from the discovery to clinical practice is still in the process with numerous limitations and pitfalls. Moreover, the biomarker validation should be confirmed and validated with huge number of specimens (48).

## Data Availability Statement

The raw data supporting the conclusions of this article will be made available by the authors, without undue reservation.

## Ethics Statement

The protocol for specimen collection was approved by the Ethics Committee for Human Research, Khon Kaen University (HE571283, HE591330, HE601149 and HE611576) and all the studies were performed in accordance with relevant guidelines and regulations. The patients/participants provided their written informed consent to participate in this study.

## Author Contributions

WL and MS contributed to the conceptualization. MS performed the methodology. JP and JVL contributed to the software. MS, WL, JP, and JVL contributed to the validation of manuscript. MS performed the formal analysis. MS, PS-n, and AT performed the investigation. WL contributed to the resources. WL and NN contributed to data curation. MS and WL contributed to writing—original draft preparation. WL, JP, PK, AW, PM, and JVL contributed to writing—review and editing. PK, ATi, NK, ATe, AJ, VV, and PS-n contributed to the visualization. WL performed the supervision. WL contributed to project administration. WL and JVL contributed to funding acquisition. All authors have read and agreed to the published version of the manuscript.

## Funding

This work was supported by an Invitation Research Grant (Grant No. IN62118), a scholarship of the Cholangiocarcinoma Research Institution (Grant No. LFCRC002/2559), a scholarship of Graduate School Khon Kaen University to MS, the National Research Council of Thailand through Fluke Free Thailand Project, the NSRF under the Basic Research Fund of Khon Kaen University through Cholangiocarcinoma Research Institute and a grant from Khon Kaen University in Thailand to WL. JVL was funded by MRC New Investigator Grant (MR/P002536/1) and the ERC Starting Grant (715662).

## Conflict of Interest

The authors declare that the research was conducted in the absence of any commercial or financial relationships that could be construed as a potential conflict of interest.

## Publisher's Note

All claims expressed in this article are solely those of the authors and do not necessarily represent those of their affiliated organizations, or those of the publisher, the editors and the reviewers. Any product that may be evaluated in this article, or claim that may be made by its manufacturer, is not guaranteed or endorsed by the publisher.
