# Supplementary material for: Metabolic Phenotyping Predicts Gemcitabine and Cisplatin Chemosensitivity in Patients With Cholangiocarcinoma
Source: Front Public Health. 2022 Feb 10;10:766023. doi: 10.3389/fpubh.2022.766023 (PMC8866176; doi:10.3389/fpubh.2022.766023)
Supplement: Supplementary file 1 [file Data_Sheet_1.pdf]

Supplementary Material

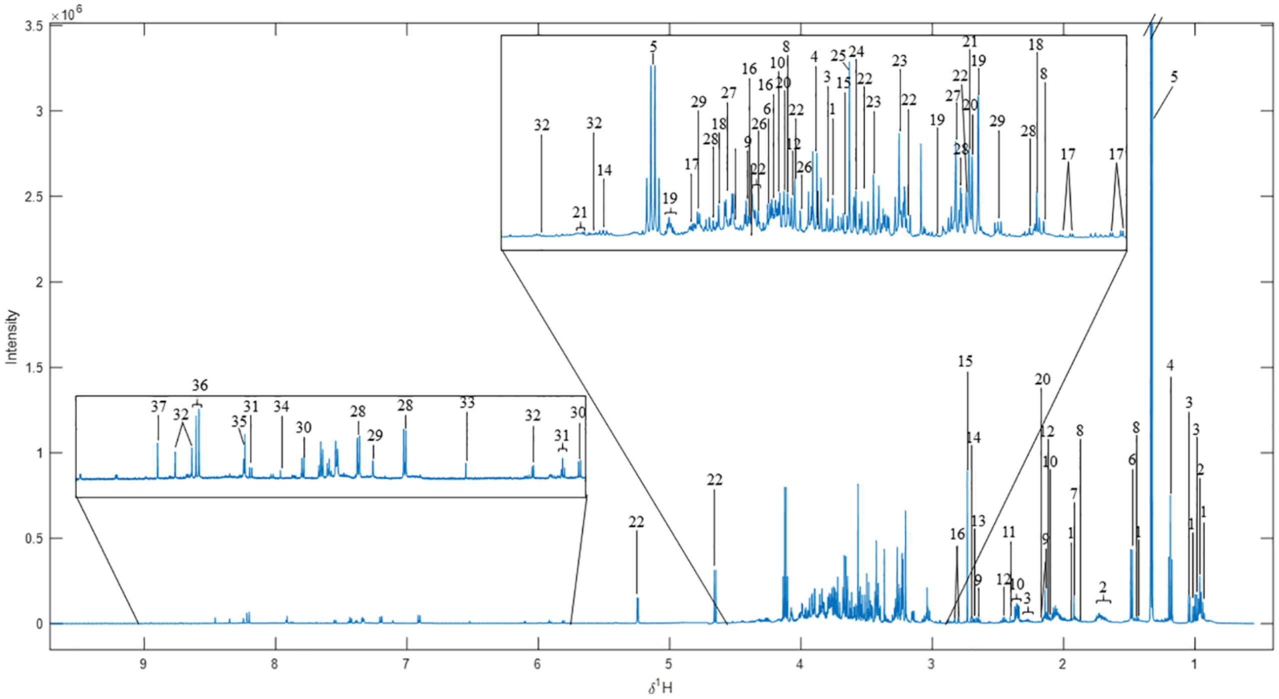

Supplementary Figure S1. Representative 600 MHz ^{1}H NMR spectra from tumor tissues of CCA patients.1 = Isoleucine; 2 = Leucine; 3 = Valine; 4 = Ethanol; 5 = Lactate; 6 = Alanine; 7 = Acetate; 8 = Lysine; 9 = Methionine; 10 = Glutamate; 11 = Succinate; 12 = Glutamine; 13 = Citrate; 14 = Malate; 15 = Sarcosine; 16 = Aspartate; 17 = Asparagine; 18 = Creatine; 19 = Choline; 20 = Acetylcholine; 21 = Glycerophosphorylcholine; 22 = Alpha-D-glucose; 23 = Taurine; 24 = Cysteate; 25 = Glycine; 26 = Glycylgycine; 27 = Betaine; 28 = Tyrosine; 29 = Histidine; 30 = Uracil; 31= Uridine; 32 = Inosine; 33 = Fumarate; 34 = Pyridoxine; 35 = Xanthine; 36 = Hypoxanthine; 37 = Formate

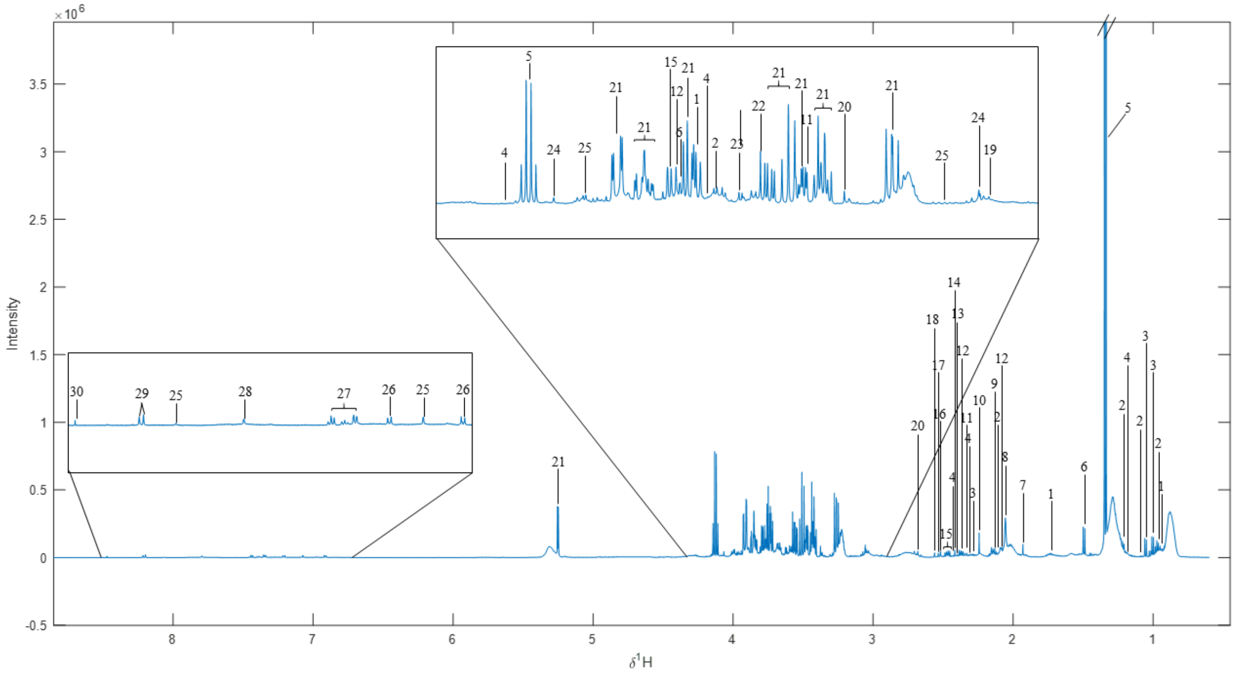

Supplementary Figure S3. Representative 600 MHz ^{1}H NMR spectra from the serum of CCA patients. 1 = Leucine; 2 = Isoleucine; 3 = Valine; 4 = Beta-hydroxybutyrate; 5 = Lactate;

6 = Alanine; 7= Acetate; 8 = Methly acetate; 9 = Trimethylamine; 10 = Pyruvic acid;

11 = Acetoacetate; 12 = Glutamate; 13 = pyruvate; 14 = succinate; 15 = Glutamine;

16 = Dimethylamine; 17 = Oxirane; 18 = Succinic acid; 19 = Creatinine; 20 = Methylguanidine;

21 = Alpha-D-glucose; 22 = Glycine; 23 = Beta-hydroxypyruvate; 24 = Histidine;

25 = Unknown; 26 = Mandelic acid; 27 = Xanthine; 28 =Adenine; 29 = Formate.

Supplementary Table S1. List of all metabolites that found in NMR spectra of tumor tissue samples.

NO.	Metabolites	Chemical shifts
1.	Isoleucine	0.9419 (t), 1.039 (d), 1.261 (m), 1.963 (m), 3.614 (d)
2.	Leucine	0.9537 (t), 1.671 (m), 3.729 (t)
3.	Valine	0.9873 (d), 1.039 (d), 2.256 (m), 3.614 (d)
4.	Ethanol	1.176 (t), 3.646 (q)
5.	Lactate	1.327 (d), 4.102 (q)
6.	Alanine	1.478 (d), 3.797 (q)
7.	Acetate	1.923 (s)
8.	Lysine	1.46 (m), 1.725 (q), 1.911 (m), 3.021 (t), 3.755 (t)
9.	Methionine	2.143 (s), 2.163 (m), 2.635 (t), 3.848 (t)
10.	Glutamate	2.105 (m), 2.34 (dt), 3.763 (t)
11.	Succinate	2.408 (s)
12.	Glutamine	2.13 (m), 2.439 (m), 3.75 (t)
13.	Citrate	2.66 (d), 2.52 (d)
14.	Malate	2.352 (q), 2.659 (q),4.295 (t)
15.	Sarcosine	2.734 (s), 3.614 (s)
16.	Aspartate	2.635 (dd), 2.798 (dd), 3.848 (dd)
17.	Asparagine	2.848 (dd), 2.939 (dd), 4.005 (dd)
18.	Creatine	3.04 (s), 3.935 (s)
19.	Choline	3.205 (s), 3.521 (m), 4.058 (m)
20.	Acetylcholine	2.164 (s), 3.22 (s), 3.74 (m),
21.	Glycerophosphorylcholine	3.231 (s), 4.312 (m)
22.	Alpha-D-glucose	3.239 (dd), 3.396 (m), 3.456 (m), 3.532 (dd), 3.72 (m), 3.82 (m), 4.648 (d), 5.24 (d)
23.	Taurine	3.258-3.28 (t), 3.416-3.438 (t)
24.	Cysteate	3.289(dd), 3.553 (dd)
25.	Glycine	3.567 (s)
26.	Glycylgycine	3.809 (s), 3.832 (s)

NO.	Metabolite	Chemical shifts
27.	Betaine	3.268 (s), 3.902 (s)
28.	Tyrosine	3.061 (dd), (dd), 3.94 (dd), 6.9 (d), 7.191 (d)
29.	Histidine	3.141 (dd), 3.248 (dd), 3.988 (dd), 7.11 (s)
30.	Uracil	5.803 (d), 7.542 (d)
31.	Uridine	5.903 (s), 5.916 (d), 7.869 (d)
32.	Inosine	4.284 (q), 4.438 (t), 6.098 (d), 8.245 (s), 8.351 (s)
33.	Fumarate	6.524 (s)
34.	Pyridoxine	7.688 (s)
35.	Xanthine	7.912 (s)
36.	Hypoxanthine	8.201 (s), 8.219 (s)
37.	Formate	8.46 (s)

S: Singlet, d: Doublet, dd: Doublet of doublet, t: Triplet, q: Quartet, m: Multiplet

Supplementary Table S2. List of all metabolites that found in NMR spectra of serum samples.

NO.	Metabolites	Chemical shifts
1.	Leucine	0.9631 (t), 1.711 (m), 3.728 (t)
2.	Isoleucine	0.9631 (t), 1.061 (d), 1.24 (m), 1.452 (m), 2.056 (m), 3.678 (d)
3.	Valine	0.9974 (d), 1.048 (d), 2.28 (dh), 3.626 (d)
4.	Beta-hydroxybutyrate	1.185 (d), 2.311 (dd), 2.413 (dd), 3.68 (s), 4.176 (dt)
5.	Lactate	1.336 (d), 4.109 (q)
6.	Alanine	1.488 (d), 3.768 (q)
7.	Acetate	1.93 (s)
8.	Methly acetate	2.056 (s)
9.	Trimethylamine	2.119 (s)
10.	Pyruvic acid	2.24 (s)
11.	Acetoacetate	2.288 (s)
12.	Glutamate	2.093 (m), 2.358 (dt), 3.783 (t)
13.	Pyruvate	2.399 (s)
14.	Succinate	2.416 (s)
15.	Glutamine	2.452 (m), 3.776 (t)
16.	Dimethylamine	2.519 (s)
17.	Oxirane	2.535 (s)
18.	Succinic acid	2.561 (s)
19.	Creatinine	3.032 (s), 4.05 (s)
20.	Methylguanidine	2.681 (s), 3.376 (s)
21.	Alpha-D-glucose	3.248 (t), 3.407 (m), 3.465 (m), 3.542 (t), 3.718 (m), 3.83 (m), 3.904 (dd), 5.248 (d)
22.	Glycine	3.575 (s)
23.	Beta-hydroxypyruvate	3.666 (s)
24.	Histidine	3.14 (dd), 3.25 (dd), 3.99 (dd), 7.073 (s), 7.793 (s)
25.	Unknown	7.202 (d), 6.907 (d)

NO.	Metabolite	Chemical shifts
26.	Mandelic acid	7.431(d), 7.341 (m)
27.	Xanthine	7.794 (s)
28.	Adenine	8.197 (s), 8.214 (s)
29.	Formate	8.472 (s)

S: Singlet, d: Doublet, dd: Doublet of doublet, t: Triplet, q: Quartet, m: Multiplet,
